# Search for additional tests for immunobiological evaluation of the candidate vaccines against African swine fever

**DOI:** 10.1371/journal.pone.0265819

**Published:** 2022-05-12

**Authors:** Alexey D. Sereda, Anna S. Kazakova, Viktor V. Dmitrenko, Denis V. Kolbasov

**Affiliations:** Federal Research Center for Virology and Microbiology (FRCVM), Volginsky, Vladimir Region, Russia; Plum Island Animal Disease Center, UNITED STATES

## Abstract

The spread of African swine fever (ASF) in Eurasia has forced a return to the development of live vaccines based on naturally or experimentally attenuated strains of the virus including those resulting from genetic manipulations. This process includes evaluation of the immunomodulating properties of the vaccines. In this report we provide our assessment of two tests for immunobiological evaluation of a candidate live vaccine against ASF from the attenuated ASF virus (ASFV) strain KK-202: (i) investigation of the effect of the attenuated ASFV strain KK-202 on the protectiveness of the vaccine ASFV strain FK-32/135 and a vaccine against classical swine fever (CSF) from the strain LK-VNIIVViM; (ii) determination of the phagocytic activity of blood neutrophils in pigs inoculated with ASFV strains differing in virulence. A simultaneous or sequential inoculation of attenuated strain KK-202 (seroimmunotype II) and vaccine strain FK-32/135 (seroimmunotype IV) into pigs resulted in the loss of protection against the virulent strain France-32 (seroimmunotype IV). Following the simultaneous or sequential inoculations of the ASFV strain KK-202 and the CSF virus (CSFV) vaccine produced from the strain LK-VNIIVViM, the neutralizing antibody titers against the CSFV observed in the experimental groups (after vaccination and after the challenge infection with the virulent CSFV strain Shimen) were not different from those found in animals of the control group. The phagocytic activity of blood neutrophils was shown to increase from 30% in the norm to 50%–94% depending on the virulence of the ASFV strains inoculated into pigs. The results of this work demonstrate the ability of the attenuated ASFV strains to modulate the development of the cellular link of protective immunity without negative impact on the humoral immune response. The informative value of the described immunobiological tests *in vivo* and *in vitro* seems to be a more preferable alternative in comparison to the commonly used *in vitro* tests, which do not always correlate with the development of protection against ASF.

## Introduction

African swine fever (ASF) is a contagious, septic disease which affects both domestic pigs and wild boars and is characterized by fever, signs of toxemia, hemorrhagic diathesis and high mortality. Depending on the virulence of the isolates, the infection can proceed in a peracute, acute, subacute, chronic or unapparent form. The ASF causative agent is the most complex DNA-containing arbovirus, and is the only member of the *Asfarviridae* family [1]. Eight *Ornithodoros* species have been demonstrated as vector competent for ASFV [2]. No ASF vaccines have been developed [3].

Classical swine fever (CSF) is a highly contagious viral disease of domestic pigs and wild boars which can proceed in an acute, chronic or subclinical form [4]. The causative agent of the disease is an RNA-containing virus belonging to the genus *Pestivirus* of the family *Flaviviridae* [5].

The ASF spread in Eurasia is forcing a return to the development of live vaccines based on naturally or experimentally attenuated virus strains including those obtained through genetic manipulations [6]. For their assessment a number of methods are being used: immunobiological test (survival of vaccinated pigs after infection with homologous virulent isolates), clinical manifestation after vaccination and challenge, determination of virus load, virus-specific antibodies in the blood, and production of IFNγ in cultures of peripheral blood mononuclear cells [7–10]. At the same time, the expediency of some of the listed criteria is debatable. For example, presence of virus specific antibodies in serum does not correlate with protection against ASFV. Assessment of the virus-specific cell mediated immunity by the level of IFNγ production is also indirect.

In addition, evaluation of the candidate vaccines requires an understanding of their possible impact on the general state of the cellular and humoral immunity in animals. Obviously, there is a need to expand variety of tests which can be used for evaluation the immunomodulatory properties of candidate vaccines against ASF.

Monocytes/macrophages and neutrophils play a key role in the development of an early antiviral response. Mediators secreted in large quantities by activated neutrophils promote maturation, differentiation, and activation of cells of innate and, in particular, adaptive immunity [11]. Neutrophils are able to migrate through the walls of blood vessels to the infected area, participate in the development of an inflammatory reaction, and implement an antimicrobial or antiviral program. The loss or decrease of these functions can be significant in the determination of the resistance or sensitivity of animals to pathogens, including secondary infections [12–17]. The currently available information on susceptibility of neutrophils to ASFV infection is contradictory. According to some data, the neutrophils are not susceptible to ASF virus [18], whereas other reports demonstrate that viral antigens, replication centers and viral particles were observed in the blood neutrophils of infected pigs [19]. The hematological acute form of ASF is characterized by neutrophilia and an increase of immature forms of neutrophils in the blood. Significant changes in the neutrophil population begin from the fourth day after infection [20].

In this report we present our experience of using two tests for immunobiological evaluation of the candidate live ASFV vaccine based on the attenuated ASFV strain KK-202 (seroimmunotype II). The first test is based on the assessment of the impact of infection with the attenuated vaccine strain KK-202 (seroimmunotype II) on protectivity of: (i) ASFV vaccine strain FK-32/135 (seroimmunotype IV), which provides protection by induction of cellular mechanisms of immunity [21, 22]; (ii) live attenuated CSFV vaccine strain LK-VNIIVViM, which provides protection by induction of humoral mechanisms of immunity (virus neutralizing antibodies (VNA)) [23]. The second test is the assessment of the phagocytic activity of blood neutrophils from pigs, inoculated with ASFV strains of different virulence.

## Materials and methods

### Animal experiments and ethics statement

In this work 45 clinically healthy pigs of a “Large White” breed 2 to 4 months old from the Experimental Animal Preparation Sector of the FRCVM, were used.

Experiments involving animals and virus were performed in accordance with the National Institutes of Health’s Guide for the Care and Use of Laboratory Animals [24] and were approved by the Bioethics Commission of the Federal Research Center for Virology and Microbiology (FRCVM) (protocol No. 6 from July 24, 2020). All experiments were performed in the Biosafety Level 3 facilities of the FRCVM, and were conducted under supervision of the institutional animal care and use and institutional biosafety committees. Animal care and procedures were performed in accordance with the guidelines of the Good Experimental Practices (GEP) and under the supervision of the Bioethics Commission of the FRCVM.

The animals were monitored twice daily for any clinical signs and to document any localized and/or systemic adverse effects. To minimize suffering and distress, were used analgesic Flexoprofen (VIK-Animal Health, Republic of Belarus) at a dose of 3 mg/kg or anesthetic Zoletil 100 (Virbac, France) at a dose of 0.025 mg/kg. It should be noted that animals developing acute form of ASFV/CSFV diseases signs were euthanized as soon as they fit: an acute form signs of ASF or CSF are: pyrexia (*˃* 41°C), refusal of feed, wobbly gait, animal lies on the side, hemorrhages on the ears and abdomen.

At the termination of the study, the animals were euthanized with a penetrating captive bolt gun meant for euthanasia purposes.

The pigs were kept and euthanized in accordance with the protocol and the Guide for the Care and Use of Laboratory Animals [24], and all efforts were made to minimize suffering.

### Viruses

The following ASFV strains were used in the research study: (i) the virulent strains Congo-49 (seroimmunotype II) and France-32 (seroimmunotype IV); (ii) attenuated strain KK-202 (seroimmunotype II), and (iii) a vaccine strain FK-32/135 (seroimmunotype IV), which were provided by the FRCVM [22]. The activity of used virus strains was of 10^7.0−7.5^ 50% hemadsorbing units (HAU_50_) per mL of stock (HAU_50_/mL). In the experiments, a dry culture viral vaccine against CSF prepared from the strain LK-VNIIVViM with an activity of 10^4.5−5.5^ 50% Tissue Culture Infectious Dose (TCID_50_) per mL of stock (TCID_50_/mL) was used [25]. To evaluate the vaccine efficacy, a virulent CSFV strain, Shimen, was used in the form of virus-containing blood with an activity of 10^6.0−6.5^ 50% Lethal Dose (LD_50_) per mL of stock (LD_50_/mL).

### Cell culture

Peripheral blood leucocytes of swine (PBLs) cells were prepared in accordance with GOST 28573–90 (2005) [26]. PBLs cell culture was prepared by the following procedure. The 30–40 mL of the pig’s whole blood were collected from the cranial vena cava to a glass tube containing heparin (20 IU/mL). The tube was placed vertically in an incubator (or in a water bath) at 37°C to allow the cells to settle down. The upper fraction consisting of plasma and leukocytes was collected and centrifugated at 1300 g for 15 min without brake. Supernatant liquid was removed and the pellet was suspended in the Eagle’s minimal essential medium (EMEM) (PanEco, Russia) (final concentration of 4x10^6^ leucocytes per mL) containing 10% of autologous serum (inactivated by heating at 56°C for 30 min), penicillin (100–200 IU/mL) and streptomycin (100–200 mg/mL). The cells were incubated in 24 or 96 well cell culture plates at 37°С in an atmosphere containing 5% CO_2_.

Continuous piglet kidney cells PK-15 were prepared in the FRCVM Cell Bank.

### Determination of the ASF and the CSF virus infectivity

The infectivity of the ASFV was determined by titration in PBLs cell culture [27]. The infectivity of the CSFV strain LK-VNIIVViM was determined by titration in the continuous PK-15 cells grown in EMEM with 2% cattle fetal blood serum, using six-well plates (Costar, France). Both the CSFV-infected PK-15 cell culture and the control were kept at 37°C in air containing 5% CO_2_ for 48 to 72 hours. At the end of incubation, the media was removed; the cell monolayer was fixed with 20% acetone in buffered saline (PBS) for 10 min at 4°C and stained with anti-CSFV IgG conjugated with fluorescein isothiocyanate (FITC). The results were read under the luminescence microscope ECLIPSE E200 (Nikon, Japan).

During the titration, the virus-containing materials were sequentially diluted 10-fold with four replicates. The virus titers were calculated according to the method of B.A. Kerber modified by I.P. Ashmarin [28].

### Determination of virus-neutralizing antibody

The immunogenicity of the CSF vaccine was evaluated by titration of blood VNA using the method of inhibition of plaques in the PK-15 cell culture [29–31]. The sera samples were serially diluted (two-fold) in 96-well plates (Costar, USA) in a volume of 0.1 mL in a growth medium containing 200 TCID_50_ of the CSFV strain LK-VNIIVViM and incubated for 1 hour at 37°C in a CO_2_ incubator. Then, 0.1 mL of PK-15 cell suspension was added to each well of the plate and incubated for 3 to 4 days. On completion, the growth medium was removed, and the cell monolayer was fixed with 20% acetone in PBS for 30 min at 4°C. Then, the cell monolayer was dried at 60°C and stained by anti-CSFV IgG conjugated with FITC. The antibody titers were expressed as 1/log_2_.

### Determination of protectiveness

The CSFV vaccine efficacy was determined by challenge of the experimental animals with the virulent CSFV strain Shimen inoculated intramuscularly at a dose of 10^4.0−4.5^ LD_50_. The efficacy of the ASFV vaccine strain FK-32/135 was determined by challenge of the experimental animals with the virulent ASFV strain France-32 inoculated intramuscularly at a dose of 10^7.0^ HAU_50_.

### Isolation and determination of the phagocytic activity of neutrophils

In total, 2.0 mL of blood was aseptically taken from the ear vein into test tubes containing 0.2 mL of an anticoagulant solution (2% sodium citrate). A gradient (55%–65%) Percoll (GE Healthcare, USA) was covered with a layer of the blood diluted with PBS (1:1) and centrifugated for 30 min at 1400 g. The neutrophil circle at the 55%–65% Percoll border was carefully collected in test tubes and washed three times with sterile PBS by centrifugation at 200 g for 10 minutes. The neutrophil content in the resulting cell suspension was about 95%, and the concentration was adjusted to 5 × 10^6^ cells/mL.

The phagocytic activity of neutrophils was determined using microscopy based on the latex particle absorption patterns (1.5 μm, DiaM, Russia). For this purpose, 100 μL of latex particles at a concentration of 10^8^/mL was washed thrice in physiological saline, and 100 μL of neutrophil suspension was mixed in nylon tubes. The samples were incubated for 30 min at 37°C; then, the contents of the tubes were resuspended, and smears were prepared which were fixed with methanol and stained according to the method of Romanowsky–Giemsa. The percentage of phagocytizing neutrophils was calculated as the number of cells that captured 1 to 10 and more than 11 latex particles from 100 phagocytes, and the phagocytosis index was derived as a ratio of the percentage of neutrophil phagocytosis as observed in the inoculated pigs to that of the intact (naïve) pigs.

### Statistics

The obtained experimental data were processed using variation statistics. For variables representing the sample collection being analyzed, an arithmetical mean value and a standard error the mean value (M ± m) were calculated.

## Results

It is known that the ASFV vaccine strain FK-32/135 at doses of 10^7.0–9.2^ HAU_50_ causes protection of up to 100% of pigs from African, European, South American isolates of the seroimmunotype IV virus in 3 to 10 days after inoculation. It has been proven that protection is formed by the effectors of cellular immunity [22, 32].

The starting point of the study described in this report was the experiment No. 1 on the effect on protection against virulent strain France-32 of the simultaneous or sequential inoculations of the attenuated ASFV strain KK-202 and/or vaccine strain FK-32/135. In total, 14 pigs were used, which were divided into five groups (No. I-V) according to the scheme represented in Table 1.

**Table 1:** The effect of inoculation of pigs in groups No. I–V with the attenuated ASFV strain KK-202 (seroimmunotype II) and/or vaccine strain FK-32/135 (seroimmunotype IV) on the subsequent challenge with the virulent strain France-32 (seroimmunotype IV).

Group No.	No.	Pig inoculation scheme	ASF viral titers in blood, log_10_ HAU_50_/mL				Death post challenge, day
			Post inoculation with vaccine strain		Post challenge		
			Day 6	Day 14	Day 6	Day 14	
I	1	KK-202 + FK-32/135 (2-day interval)	2.00	1.50	3.75	3.75	18
	2		2.50	2.50	3.00	3.50	29
	3		2.75	1.75	3.25	3.50	27
II	4	KK-202 + FK-32/135 (simultaneous)	3.50	3.00	3.75	6.75	21
	5		3.25	3.00	3.50	6.00	27
	6		3.25	3.00	3.50	6.25	27
	7		3.50	3.25	3.25	5.50	29
III	8	FK-32/135	<0.25	n.d.*	1.00	n.d.	S***
	9		<0.25	n.d.	1.50	n.d.	S
	10		<0.25	n.d.	1.00	n.d.	S
IV	11	KK-202	3.00	4.25	6.50	†**	8
	12		3.50	3.75	6.25	†	8
	13		3.50	4.75	7.00	†	10
V	14	Control	-	-	6. 75	†	9

Note: n.d.*—no virus is detected in blood; †**—died; S***—survived.

The ASFVs were inoculated to pigs intramuscularly: strain KK-202 at a dose of 10^7.5^ HAU_50_, and strain FK-32/135 at a dose of 10^7.0^ HAU_50_. Fourteen days later, the animals were challenged by intramuscular inoculation with the virulent ASFV strain France-32 (which belongs to the same seroimmunotype IV as the strain FK-32/135) at a dose of 10^7.0^ HAU_50_.

**Fig 1:**
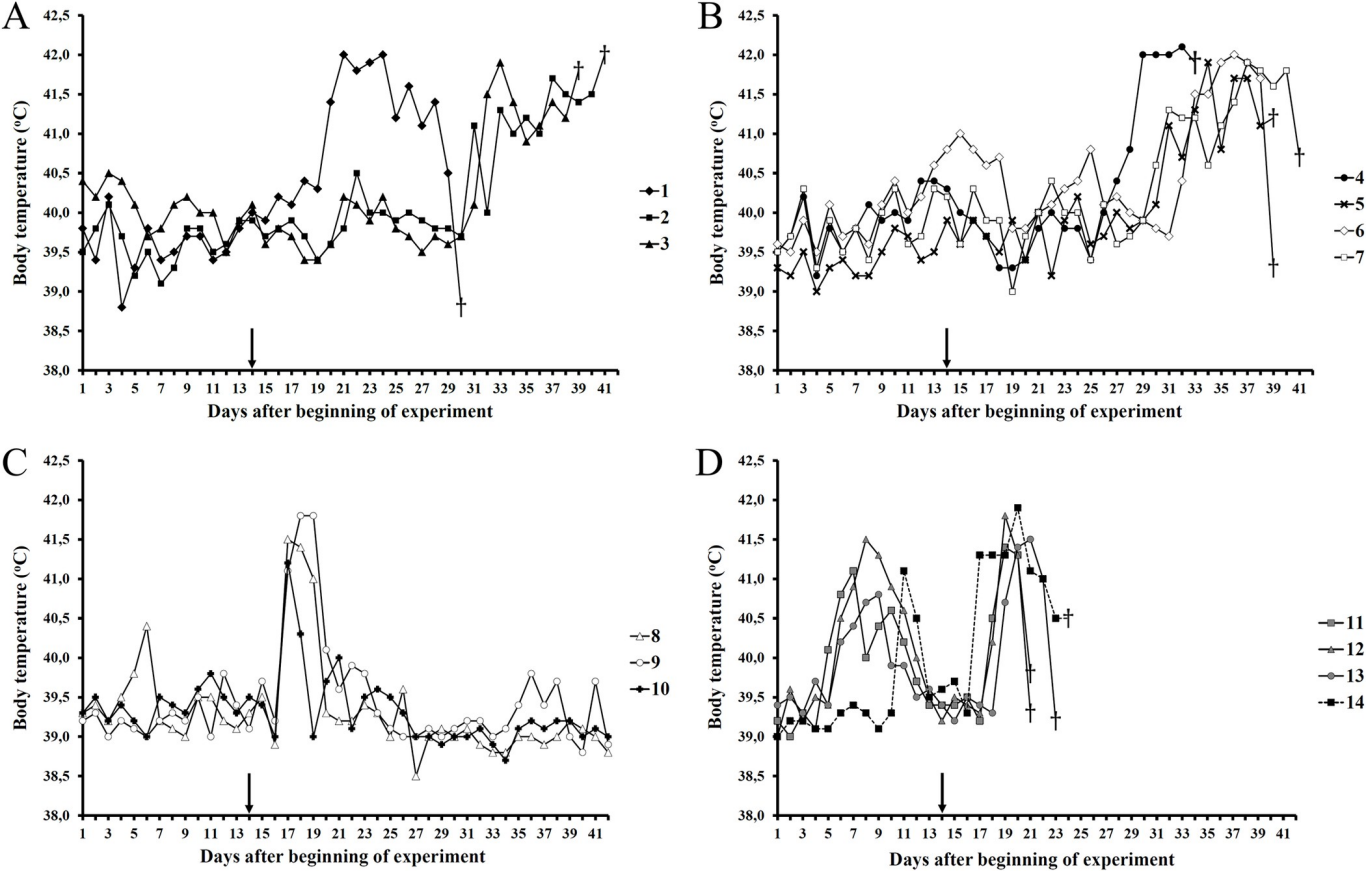
The kinetics of body temperature values of pigs inoculated with attenuated ASFV strain KK-202 (107.5 HAU50) and/or vaccine strain FK-32/135 (107.0 HAU50) and subsequently challenged with the virulent ASFV strain France-32 (107.0 HAU50) on day 14. A. (1–3) KK-202 + FK-32/135 (at an interval of 2 days); B. (4–7) KK-202 + FK-32/135 (simultaneously); C. (8–10) FK-32/135; D. (11–13) KK-202 and (14) naïve (control). Each curve represents the values of individual animals in the groups.

Pigs from groups No. I (see Table 1 and Fig 1A) and No. II (see Table 1 and Fig 1B) fell ill and died between 16 to 27 days post challenge infection, all the animals from group No. III (see Table 1 and Fig 1C) survived after short-term hyperthermia from 3 to 5 days post infection and those of group No. IV (see Table 1 and Fig 1D (animals # 11–13)) and control group No. V (see Table 1 and Fig 1D (animal # 14) died in the period from 7 to 9 days post infection.

Viremia indices on day 6 after challenge and the timing of death of animals from groups No. IV and No. V (control group) coincided with previously published data on the immunobiological properties of the France-32 virulent strain [32].

The low levels of viremia in animals of group No. III on day 6 after infection with the virulent strain France-32 and the absence of the virus in blood on day 14 indicated that protection against ASFV of a homologous seroimmunotype has been induced. The high levels of viremia in animal groups No. I and No. II on days 6 and 14 post infection and their subsequent death were interpreted as a consequence of the immunomodulation caused by the inoculation of the animals with the attenuated strain KK-202.

In experiment 2, the effect of sequential or simultaneous inoculations of 11 pigs with the ASFV strain KK-202 at a dose of 10^7.5^ HAU_50_ and/or the CSFV vaccine from strain LK-VNIIVViM at a dose of 10^3^ TCID_50_ were studied. There were three pigs in each group (group No. 1, 2, and 3), except group No. 4, which had two pigs (Table 2). The clinical condition of animals, titers of VNA against CSF and protection against virulent CSFV strain Shimen were evaluated.

**Table 2:** The titers of virus-neutralizing antibodies against CSFV in pigs inoculated by attenuated ASFV (KK-202) and vaccine CSFV (LK-VNIIVViM), and results of the challenge on day 28 with the virulent CSFV strain Shimen.

Group No.	Pig inoculation scheme	VNA titers (1/log_2_) post			Diseased/total post		Died/total
		Vaccination		Challenge	Vaccination	Challenge	
		Day 14	Day 28	Day 42			
1	KK-202+LK-VNIIVViM (3-day interval)	4.5±1.5	7.0±1.2	8.7±0.3	3/3	0/2	1/3*
2	KK-202+LK-VNIIVViM (simultaneous)	5.7±0.9	6.0±1.0	9.0±0.0	3/3	0/3	0/3
3	LK-VNIIVViM	3.7±1.2	6.7±0.9	9.0±0.8	0/3	0/3	0/3
4	Intact (naïve)	-	-	-	-	2/2	2/2

Note: *—the animal died prior to the infection with the virulent CSFV strain Shimen.

The design of the experiment and the main results summarized in Table 2 suggest that the titers of neutralizing antibodies against CSFV determined in groups No. 1 and 2 were not different from those observed in animals of the control group No. 3, both after the vaccination and following the challenge by the virulent CSFV strain Shimen.

The clinical condition of pigs from group No. 3 throughout the experiment remained stable and did not differ from that before vaccination and challenge. Pigs from groups No. 1 and 2 became ill (body temperature from 40.2 to 40.9°C, depression) in the period from day 6 to 10 after the start of the experiment. One pig from group No. 1 died on day 16. After infection with the virulent CSFV strain Shimen both pigs from group No. 4 fell ill on days 6–7 and died on days 14–15, whereas eight pigs from groups No. 1–3 did not show any clinical signs of the disease.

**Fig 2:**
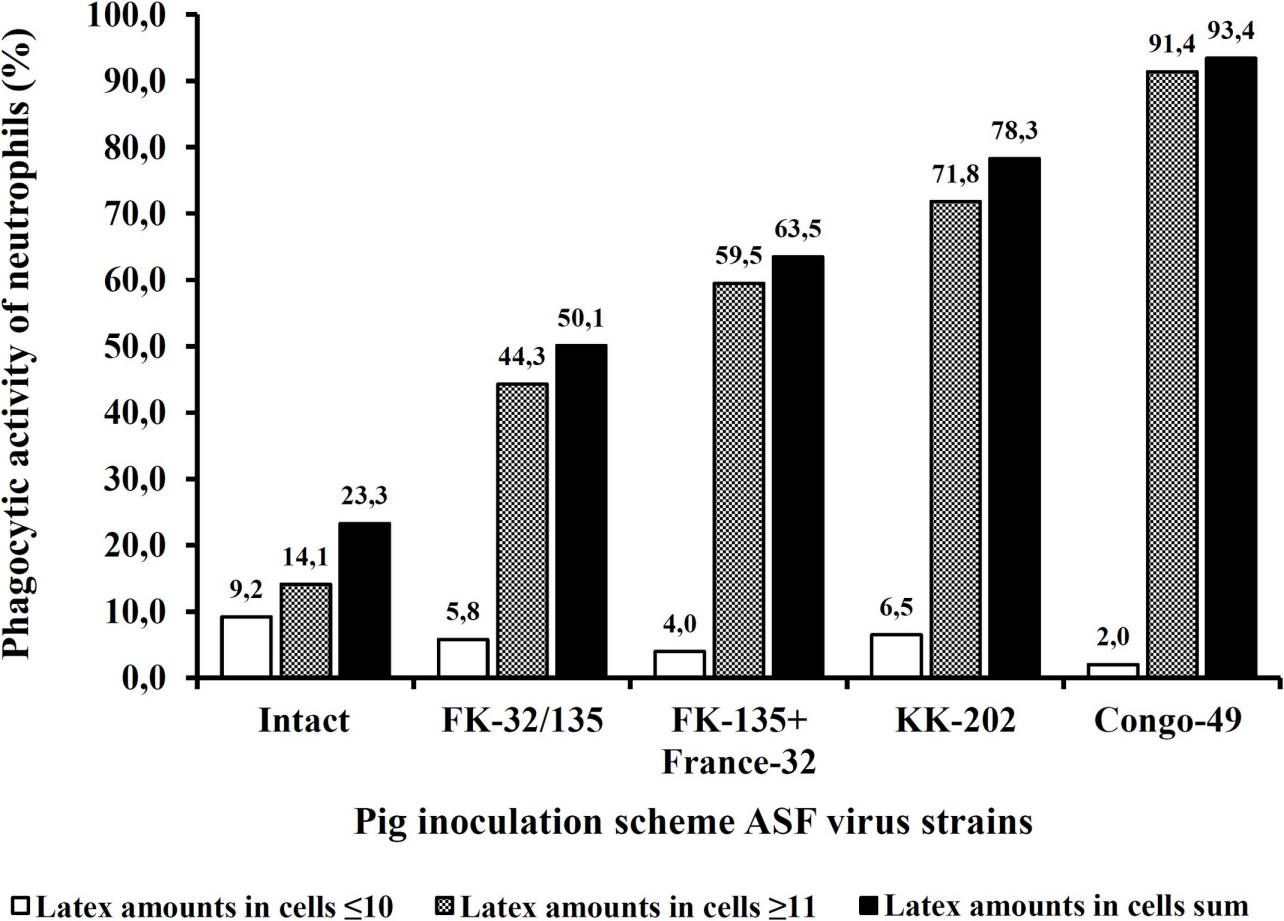
The effect of pig inoculation with attenuated, vaccine and virulent ASFV strains on the mean phagocytic activity of porcine blood neutrophils (blood samples were collected four days after inoculation), n = 4.

The functional activity of blood neutrophils was examined in 20 pigs (4 pigs per group) inoculated with attenuated strain KK-202, or vaccine strain FK-32/135, or virulent strain Congo-49, or with FK-32/135 and subsequent infection with virulent strain France-32 at doses of 10^7.0−7.5^ HAU_50_, following the scheme of the experiment 3 given in Fig 2. The phagocytic activity of neutrophils was examined on day 4 post inoculation. In all of the animal groups an increase in phagocytic activity and changes in morphology of blood neutrophils were recorded. The vaccine strain FK-32/135 caused a relatively moderate (compared to intact animals) increase of the phagocytic capacity of blood neutrophils (n = 4, P<0.05). The phagocytosis of latex particles was observed with 50.1% of the cells, and about 44.3% of neutrophils had a high specific activity (i.e., they absorbed ≥11 particles). Morphologically, the structure of the latex-absorbed neutrophils was subjected to some alterations: the cells swelled with no membrane integrity damage. The clinical conditions of animals inoculated with strain FK-32/135 and intact animals were the same at the time of assessment of the blood neutrophils phagocytic activity.

The increased phagocytic activity of neutrophils was observed in pigs inoculated with the attenuated strain KK-202 and the highly virulent strain Congo-49. The percentages of cells that had absorbed latex were 78.3% and 93.4%, respectively. The phagocytic activity of blood neutrophils from pigs inoculated with the non-reactogenic vaccine strain FK-32/135 following by infection with the virulent strain France-32 4 days after was moderate.

## Discussion

Despite the understanding of the main principles of the formation, action and mechanisms of protection, no commonly acceptable laboratory tests for evaluation of the candidate ASFV vaccines have been established. We believe that it is necessary to expand the share of laboratory tests characterizing the functional activity of the immune system, since they can objectively reflect the real effect of vaccines on animals. In this study we evaluated the candidate live ASFV vaccine (strain KK-202) against ASFV of seroimmunotype II using two tests: (i) determination of the protectivity of the vaccine strains FK-32/135 (ASFV seroimmunotype IV) and LK-VNIIVViM (classical swine fever virus (CSFV)) on the background of the infection with KK-202, and (ii) comparative evaluation of phagocytic activity of blood neutrophils of pigs inoculated with ASFV strains of different virulence. Selection of these vaccine viruses (ASFV FK-32/135 (seroimmunotype IV) and CSFV LK-VNIIVViM) is determined by the opposite immunobiological mechanisms of protection associated with ASFV and CSFV infections. The protection against ASFV infection is associated predominantly with the cell-mediated immune response, especially antibody-depended cellular cytotoxicity (ADCC), and activity of cytotoxic T lymphocytes (CTL). The protection against the CSFV infection is based predominantly on the humoral immune response (neutralizing antibodies) [33–35].

We found that consecutive or simultaneous inoculation of pigs with the attenuated ASFV strain KK-202 and vaccine strain FK-32/135 led to disruption of the protective properties of the latter. As a result, the strain FK-32/135 had lost its ability to induce protection against virulent ASFV strain France-32 of homologous seroimmunotype. This data suggests that some attenuated strains, which are considered as candidate live ASF vaccines, can disrupt the development of antiviral immunity mechanisms aimed at elimination of the cells infected with ASFV.

The simultaneous or sequential administration of the vaccine prepared from the strain LK-VNIIVViM and the attenuated strain KK-202 carried out in our experiments did not affect the immunogenic or protective properties of the vaccine against CSF. Titers of VNA and the results of challenge with the virulent strain Shimen in experimental groups No. 1 and No. 2 did not differ from the control group No. 3, which was inoculated only with the LK-VNIIVViM vaccine. At the same time, the immunomodulatory properties of the CSFV vaccine, apparently, have promoted an increase of reactogenicity of the ASFV strain KK-202 if this vaccine was inoculated sequentially or simultaneously with the CSFV vaccine. All six pigs from groups No. 1 and 2, inoculated with these two vaccine strains, showed clinical symptoms of the disease, with one pig (from group No. 1) succumbed to the infection. Taking into account high and similar VNA titers to the CSFV in all animals which were immunized with the CSFV vaccine strain (from groups No. 1, 2 and 3), it was concluded that the disease developed due to the attenuated strain KK-202 (from two groups: No. 1 and No. 2).

Currently, the functional parameters of neutrophils, which have an important role in the defense against bacteria and viruses, have not yet been fully investigated. In our experiments in domestic pigs, we demonstrated that the increase of the virulence of the ASFV strains had resulted in an increase of the phagocytic activity of the blood neutrophils. Noteworthy is the fact that the phagocytic activity of blood neutrophils in pigs inoculated with the attenuated KK-202 strain exceeds that in pigs vaccinated with the FK-32/135 vaccine strain and subsequently challenged with the France-32 virulent strain. This feature negatively characterizes the attenuated KK-202 strain as a vaccine candidate. Scientific publications as well as our results do not allow us to assume that there is a connection between the phagocytic activity of neutrophils and protection against ASF. We report only a fact that an increase of the functional (phagocytic) activity of blood neutrophils correlates with an increase of the pathogenicity of ASF virus strains inoculated in pigs. We believe that this simple test can be used for characterization candidate live ASF vaccines both: after vaccination, and after control infection with a virulent strain.

Attenuated ASFV strains are known to cause the development of a chronic form of the disease. This form was observed on a massive scale in pigs vaccinated with a Portuguese ASFV isolate attenuated by sequential passages in primary blood macrophages (PBM) cell cultures [36]. In approximately 25% to 47% of animals inoculated with a naturally attenuated virus isolate ASFV/NH/P68, a disease with the chronic lesions, long-term fever, viremia and high levels of the antiviral antibodies with severe hypergammaglobulinemia had been observed [37]. Some immunopathological conditions including hypergammaglobulinemia and systemic immune activation were observed in pigs inoculated with other moderately virulent ASFV isolates [38, 39]. The less pronounced clinical reactions such as fever and joint swelling had been described for the ASFV strain OURT88/3 [40, 41].

## Conclusion

The results of this work demonstrate that the attenuated ASFV strains can modulate cellular immune response but has no impact on the humoral immune response. We obtained the first evidence of the ability of attenuated ASFV strain to suppress the protectivity of the vaccine ASFV strain of another seroimmunotype. The test for measuring the phagocytic activity of pig blood neutrophils, in our opinion, deserves attention as an additional test for characterization of the candidate ASFV vaccine strains.
